# Regulation plays a multifaceted role in the retention of gene duplicates

**DOI:** 10.1371/journal.pbio.3000519

**Published:** 2019-11-22

**Authors:** Johan Hallin, Christian R. Landry

**Affiliations:** 1 Département de biochimie, microbiologie et bio-informatique, Faculté des sciences et de génie, Université Laval, Québec, Canada; 2 Département de biologie, Faculté des sciences et de génie, Université Laval, Québec, Canada; 3 Institut de Biologie Intégrative et des Systèmes (IBIS), Université Laval, Québec, Canada; 4 PROTEO, Le réseau québécois de recherche sur la fonction, la structure et l’ingénierie des protéines, Université Laval, Québec, Canada; 5 Centre de Recherche en Données Massives (CRDM), Université Laval, Québec, Canada

## Abstract

A gene duplication can lead to all sorts of problems in a cell. However, it can also lead to all sorts of benefits. Beneficial or not, the gene duplicates might be kept in the genome because of several different reasons. For instance, if natural selection works towards optimizing one function of a gene at the expense of another, then gene duplication could resolve this conflict by separating the functions in two genes. Here, we outline evolutionary incentives to keep a duplicated gene in the genome, focusing on divergence in expression and trade-off resolution as featured in a new and exciting paper published in this edition of *PLOS Biology*.

## Abstract 2

Gene duplication can lead to problems for a cell, but can also afford a range of opportunities. This Primer explores the evolutionary incentives to keep a duplicated gene in the genome, focusing on a recent demonstration that duplication can help resolve conflicts in gene regulation.

Genes have life cycles of their own. They are, most of the time, born from the duplication of other genes (Box 1) and may eventually die and become pseudogenes. During the period between birth and death, the sequence and regulatory elements of a new gene change through mutations. This dynamic gain and loss of genes and the associated changes to regulation and function contribute to phenotypic differences between species and among populations of the same species [1]. Numerous studies over the past 50 years have investigated the role of different evolutionary forces such as natural selection and drift in shaping these life cycles, for example, by investigating the contribution of nucleotide and amino acid substitutions to the divergence of new genes. One category of molecular changes that appears to play a key role in the evolution of genes that originate from gene duplication (duplicates or paralogs) are regulatory changes, i.e., changes in the gene itself or elsewhere in the genome that determine when, where, and at what level a gene is transcribed and translated.

**Box 1**

Gene duplicates originate mainly by two mechanisms: small-scale duplication (SSD) and whole-genome duplication (WGD) [30]. In SSDs, only one or a few genes are duplicated, whereas in WGD, all genes are duplicated simultaneously. These two mechanisms have specific features that influence the retention of duplicates, which in turn influences the properties of genes that originated from either mechanism. One of the key differences is that SSD genes first originate in a single individual and must increase in frequency by drift or selection to be maintained. WGD would also occur in one individual, but it could potentially incite or co-occur with a speciation event [31], which would coincide with a population bottleneck and thus the fixation of all duplicates without the need for natural selection. However, WGDs have been associated with performance traits in plants, for instance [32], which means natural selection can also favor their fixation.

There is a major difference between SSDs and WGDs if we consider interactions among gene products—for instance, for proteins forming protein complexes. WGD will likely maintain the stoichiometric balance of the complexes, whereas the duplication of a single subunit through SSD would perturb the balance [33]. In a WGD, this principle predicts the preferential maintenance of proteins that are dosage sensitive and whose loss would lead to a fitness defect because it would perturb the balance. WGD genes being more dosage-balance sensitive [34] is supported by observations that they have fewer copy-number variations in human populations [35] and are overrepresented among genes with copy-number variations that are pathogenic [36]. The properties of genes may therefore influence the probability that their duplicates are maintained after SSDs or WGDs, thereby determining what is the extent of novelties that can evolve from these mechanisms. In the case of Chapal and colleagues [25], the Msn duplicates show a fitness trade-off when expression is increased and are thus dosage sensitive, which suggests that their duplication may initially have been maintained specifically because it originated via a WGD.

## Regulatory evolution and the maintenance of gene duplicates

**Fig 1:**
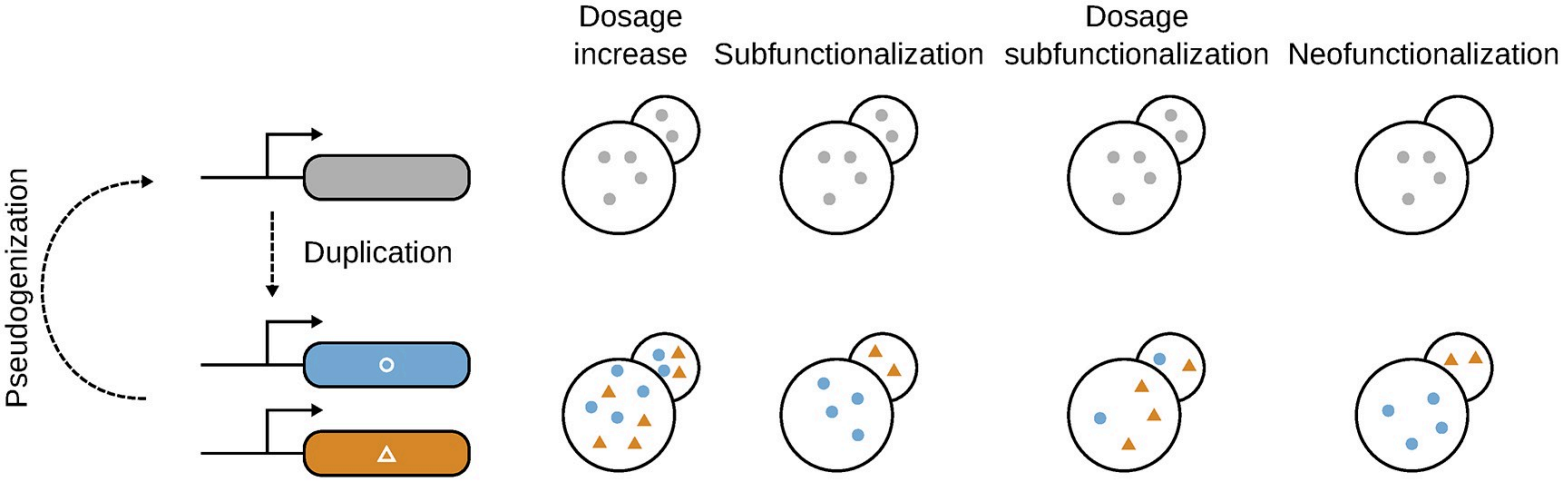
Regulatory evolution in the retention and divergence of gene duplicates. The cells represent a mother and a daughter cell to illustrate different phases of development. The top row shows these cells between the duplication event and the divergence of the duplications. The same concept applies to multicellular organisms with different cell types. Colored dots represent gene products from the ancestral gene (gray, in the cells at the top) and from the duplicated genes (blue circles and orange triangles, in the cells at the bottom). Retention by dosage effects refers to a gain in fitness caused by a larger amount of gene product. The dosage change does not need to correspond to an exact doubling (as illustrated) but could be higher [2] or lower than that if, for instance, some mechanisms of attenuation are present [3]. Retention by subfunctionalization refers to the maintenance of the two copies by the splitting of the ancestral function, here illustrated by the different localizations. Dosage subfunctionalization refers to a special case in which the total expression is maintained, but the abundance of each duplicate can change. Neofunctionalization refers to the evolution of a new function by a paralog, here shown by the new localization. In the absence of sufficient selection pressure to maintain two copies, the system can revert to a single gene system through pseudogenization (or simple loss by other mechanisms) of one copy.

The immediate effect of gene duplication is typically an increase in gene dosage [4] (Fig 1). Higher dosage, however, does not always translate into increased fitness [5]. This means that at this stage, natural selection could favor gene retention or loss, or if the expression change is effectively neutral, the duplicate could evolve neutrally for extended periods of time (Box 1). If an increase in total expression is favored by internal or external conditions, a gene duplication could provide an immediate benefit. For example, genes coding for digestive enzymes, such as amylases that hydrolyze starch, vary in copy number among human populations. Copy-number correlates with the diet such that high-starch diets are associated with more copies, whereas low-starch diets are associated with fewer copies [6]. Diet is such a strong selective force that multiple copies have been maintained in many mammals [7]. Selection for higher dosage can sometimes lead to the maintenance of a large number of gene copies. An extreme example of this is the hundreds of duplicated copies of ribosomal RNA genes in certain microbial genomes: the adaptiveness of this most likely derives from some life-history traits that demand an increase in protein synthesis machinery [8]. When paralogs are maintained because of dosage effects, gene copies are maintained without the need for the individual copies to gain new functions.

Regulatory evolution could also favor the retention of a new gene by changing the tissue or timing of expression, a process called neofunctionalization (Fig 1). The duplicate’s newly gained expression pattern would favor its retention by contributing a new function to, for instance, a tissue. The gain of new expression specificity can also be accompanied by and facilitate the gain of new molecular functions at the protein level. Indeed, the change of cellular context for the protein can represent new opportunities for selective forces to act on the protein itself. The retinoic acid receptors (RARs) have evolved following this path in vertebrates. RARs are nuclear receptors that are bound by specific ligands and that activate the transcription of genes during key developmental steps. Three paralogs originated from the whole-genome duplications (WGDs) at the origin of vertebrates, two of which have evolved new ligand-binding specificities and expression patterns during development [9].

Although it is easy to conceive that natural selection may favor the maintenance of gene duplicates because of dosage effects or new regulatory programs, it may be less intuitive to imagine that gene duplicates could be maintained by degenerative mutations that lead to the “specialization” of each duplicate (subfunctionalization, Fig 1). The theory behind the role of this mechanism was formally derived by [10,11]. Briefly, the model showed that if a gene has multiple functions or tissues of expression, its duplication could be followed by the loss of different functions in each copy while still preserving all the ancestral functions. However, because now the two genes are required to perform the functions previously performed by the single progenitor, natural selection will act to maintain both copies. The power of this model is that it does not require the evolution of new and adaptive functions, which may be inaccessible for many genes and thus could not explain why gene duplicates would be maintained. Regulatory subfunctionalization was recently hypothesized to occur at the level of alternative splicing and subcellular localization of the plastid ascorbate peroxidase in plants, a key detoxifying enzyme. Some plants have a single gene that produces two distinct proteins by alternative splicing that localize in different cell compartments; others have two independent genes, each producing a single protein that localizes to one compartment or the other [12].

Recent work showed that subfunctionalization could also take place at the level of gene dosage (dosage subfunctionalization, Fig 1) and not necessarily implying the loss of other molecular functions such as tissue or timing expression specificity. Gout and Lynch [13] showed that natural selection to maintain the expression level of a gene could act on total expression of a gene pair rather than on each of them individually. This allows the two genes to accumulate mutations that change the expression levels without being filtered by natural selection, if total expression is maintained. For instance, if one copy accumulates mutations that reduce expression, then the other copy could accumulate mutations that increase expression, all the while maintaining the total expression level. Eventually, one copy could be expressed at such a low level that its loss would be effectively neutral.

## Other dimensions of gene expression regulation

The examples mentioned thus far consider only a few dimensions of gene regulation (level, timing, localization). Yet gene expression systems are highly dynamic, and other features may contribute to the evolution and retention of gene duplicates. Two important expression features are (1) the responsiveness, which refers to the magnitude and propensity to change gene expression levels in response to intra- and extracellular signals, and (2) expression noise. Responsiveness has mostly been studied in single-celled organisms such as yeast, in which expression level has been studied in entire populations across hundreds of growth and stress conditions as well as at the single-cell level using various reporters. There are important differences in the sensitivity of genes to environmental changes and mutations: some genes rarely change expression levels, whereas others do so easily [14]. Interestingly, responsiveness also appears to correlate with divergence of gene expression levels among species [15]. More responsive genes show more differences in expression regulation between species. This observation suggests that responsiveness could be a gene property that favors divergence between species. However, this is not a universal trend, as responsiveness could also be selected against for many genes that would rather require stable dosage [16].

Expression noise appears to be strongly associated with responsiveness. Noise is linked to the architecture of the genes themselves and is manifested by expression differences among cells that are genetically identical. Noisiness is not always easily assessed because it requires cells to be examined individually. Although some studies have suggested that noise in gene expression could be advantageous [17], it is also likely to be deleterious because it prevents a large fraction of a population from attaining the optimal expression level at a given time [18]. Attesting to the importance of low noise, essential genes and (most importantly) those that reduce fitness when their dosage is reduced (haploinsufficient) tend to be less noisy than genes that do not show measurable effects on fitness upon deletion [19,20]. Furthermore, the study of the fitness consequences of noise and changes in average expression has revealed that noise could, in some cases, be as detrimental as changes in mean expression [21,22].

Nevertheless, gene expression noise is prevalent. This prevalence could be explained by the fact that for some gene classes and promoter architectures (those with a TATA box), responsiveness and noise seem to be intrinsically coupled [23]. Although natural selection may favor responsiveness, the inability of cells to reach a precise expression level comes as an unavoidable cost. This correlation between responsiveness and noise was detailed by Lehner [24], who also suggested that this trade-off could be alleviated by gene duplication because it would allow the system to maintain responsiveness while reducing noise. If the deviation from optimality of expression level of the two genes is not correlated, their average expression will be closer to optimal level than the expression of an individual gene with the same average expression and noise level. Gene duplication in this case would allow for two responsive genes but with reduced absolute noise. The consideration of gene regulation at the single-cell level therefore allowed geneticists to uncover potential mechanisms for the maintenance of gene duplication. However, a detailed example of what role these features of gene expression play in the maintenance of duplicates was yet to come.

## Single-cell biology offers a new perspective on the role of regulatory evolution in the retention of gene duplicates

This edition of *PLOS Biology* [25] brings forth an elegant example of a gene duplication that did not result in neofunctionalization or subfunctionalization as typically defined. The transcription factor Msn was duplicated during the WGD in the budding yeast *Saccharomyces cerevisiae* and has since diverged into Msn2 and Msn4. Previous studies of these two genes have differed in their conclusion as to the divergence of function between them. Despite previous suggestions that these two transcription factors may have diverged in terms of function [27], Chapal and colleagues provide convincing evidence that they regulate the same target genes. That raises the question: How and why would yeast have kept these paralogs for the last 100 million years?

**Fig 2:**
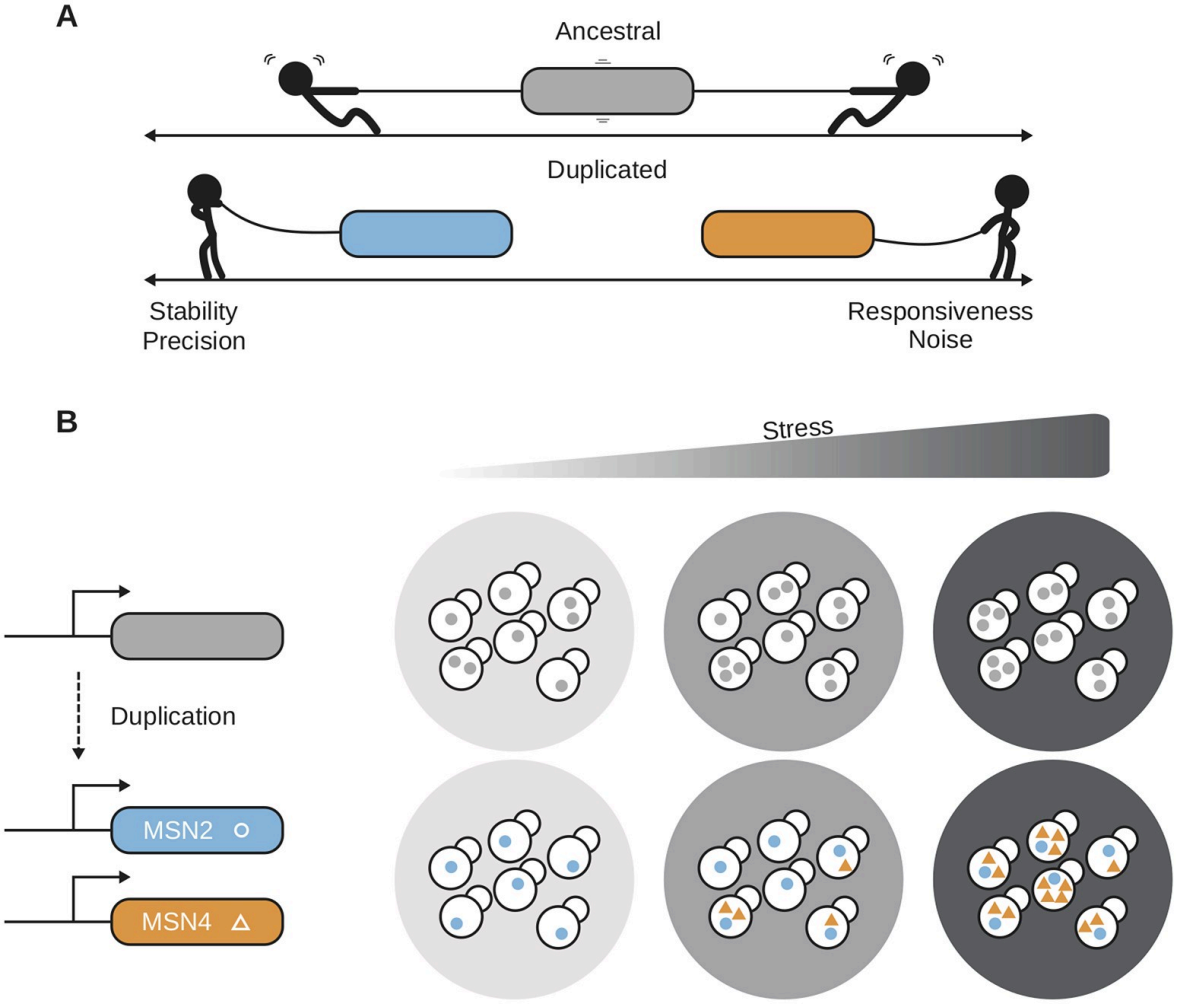
Gene duplication as a force for trade-off resolution. (A) Nonduplicated genes are forced to sacrifice expression responsiveness for precision because of the noise that accompanies responsiveness. Gene duplication can resolve this conflict by providing two genes with the same function at the protein level, one that is responsive and one that has a precise expression level. (B) Chapal and colleagues [25] provide a description of how the paralogs Msn2 and Msn4 cooperate to supply the cell with a precise but stable (Msn2) and responsive but noisy (Msn4) expression. Msn4 is hardly expressed during nonstress conditions but increases in expression as stress levels go up, whereas Msn2 remains constantly expressed at a low level. *Panel A inspired from* [26].

Chapal and colleagues bring forward compelling evidence that these two transcription factors are cooperating in the cell to minimize growth speed defects while maximizing stress responsiveness. The authors show that higher levels of Msn2 are detrimental to the growth of the cells but beneficial when cells are in stressful conditions. They propose this simple trade-off between growth speed and environmental responsiveness as the incentive for the retention of the two copies of Msn, even though they have the same target genes. In accordance with this, Chapal and colleagues [25] found that Msn2 has a low but steady expression with little noise, whereas Msn4 is environmentally responsive with a high level of noise. This allows for a regulatory dynamic that solves the conflict between a dynamic response, which comes with the trade-off of noisy expression, and a steady number of proteins in the cell during nonstress conditions (Fig 2). The expression of Msn2 does not change during the growth of a population, whereas Msn4 increases gradually along the growth curve and with it, the resistance to stress.

Interestingly, they compare the expression of the two paralogs with an ortholog from *Kluyveromyces lactis*, which diverged from *S*. *cerevisiae* before the WGD event (Box 1), and found that it had an expression profile that was intermediate to that of Msn2 and 4. The ortholog was induced throughout the growth curve, although at a lower level than Msn4, and its noisiness was intermediate between the two (Fig 2B). The authors suggest the following scenario: After the WGD, Msn2 gained a more stable expression by its transcription start site moving farther away from the open reading frame to the boundary of a nucleosome free region. Msn4, on the other hand, increased its dynamic range and noise by gaining new transcription factor binding sites.

The model proposed by this new study [25] is not, strictly speaking, about a case of subfunctionalization, because the initial model by Lynch and colleagues [10,11] does not require that the division of labor occurs with a gain in fitness. The case documented here rather suggests that division of labor allows for a gain in fitness by resolving a trade-off, as has been proposed for other pairs of paralogs that may have conflicting protein functions [28,29]. It is unclear how frequent this form of adaptive subfunctionalization is, given that many more types of conflicts may exist between the different functions of a given gene and may not necessarily be resolvable by simple mutational events. All cases of putative subfunctionalization may need to be dissected in detail as Chapal and colleagues did, to make sure that what appears to be a simple division of labor may not be accompanied with an exquisite functional specialization.

## Abbreviations

RAR: retinoic acid receptor
SSD: small-scale duplication
WGD: whole-genome duplication
